# Text Book of Medical Jurisprudence and Toxicology

**Published:** 1884-12

**Authors:** 


### Book Reviews.

Text Book of Medical Jurisprudence and Toxicology. *By* John J. Reese, m. d., *Professor of Medical Jurisprudence and Toxicology in the University of Pennsylvania. 8vo., pp. xii., 606. Philadelphia:* P. Blakiston, Son & Co., 1884.

For a long time the medical profession has been well supplied with excellent treatises on Toxicology and Jurisprudence, but among them there has been no work really suitable as a text book for American students.

The large works in French and German have either been inaccessible, or out of the question because of their size, and while in English, there has been no dearth of good material, it has not been in a form that could be used as a text book to accompany a course of lectures as given on this topic in our best schools.

Professor Reese has lately written a work which will fill out this gap in our literature, which, while not pretending to the completeness or thoroughness of such treatises as Woodman and Tidy's Forensic Medicine and Toxicology, or the author's own work on Toxicology, is yet full enough for the ordinary needs of the student, and covers the ground he should know.

That a medical practitioner should be acquainted with the details of toxical analysis is not to be expected, nor is it at all desirable, as this is a specialty which should be left to the most thoroughly trained chemists. But in relation to medical jurisprudence and those parts of toxicology which pertain to diagnosis of poison cases, he stands in a somewhat different light, and time spent, in obtaining a good knowledge of this subject, is well spent.

Dr. Reese's book does not burden the subject with the minute points of chemical examination, but the information on other matters is as full and broad as could be expected in a college text book. Good paper and clear print are important points, which the publishers have not overlooked.

